# The complete mitochondrial genome of black sea cucumber *Holothuria leucospilota* (Aspidochirotida holothuriidae)

**DOI:** 10.1080/23802359.2019.1673250

**Published:** 2019-10-04

**Authors:** Shengping Zhong, Yonghong Liu, Lianghua Huang, Yanfei Zhao, Guoqiang Huang

**Affiliations:** a Institute of Marine Drugs, Guangxi University of Chinese Medicine, Nanning, China;; b Key Laboratory of Marine Biotechnology, Guangxi Institute of Oceanology, Beihai, China

**Keywords:** Mitochondrial genome, *Holothuria leucospilota*, Holothuroidea

## Abstract

The black sea cucumber, *Holothuria leucospilota*, is an ecologically and economically important holothuroid in China due to broader environmental adaptation and valuable pharmacological compounds. However, the wild stocks of *H. leucospilota*, have been declining steadily due to overexploitation in recent years. The stock enhancement programme has become an increasingly important priority. In this study, we report the complete mitochondrial genome of *H. leucospilota*. The mitogenome has 15,839 base pairs (57.6% A + T content) and made up of total of 37 genes (13 protein-coding, 22 transfer RNAs and 2 ribosomal RNAs), and a putative control region. The complete mitogenomes of *H. leucospilota* will provide useful genetic information for future conservation and management of this valuable and vulnerable species.

The sea cucumber family Holothuriidae is the most diverse group in the Holothuroidea class and it contains about 200 valid in five nominal genera, which is the most valuable and vulnerable inshore fisheries resources (Uthicke et al. 2009; Borrero-Pérez et al. 2010). The black sea cucumber *Holothuria leucospilota*, widely distributed throughout the tropical Indo-west Pacific region, is an ecologically and economically importance species in China which was known as a sediment transporter and contains a variety of biological and pharmacological compounds (Huang et al. 2018). However, despite its high diversity and wide distribution, the wild stocks of *H. leucospilota*, have been declining steadily due to overexploitation recently. The management of *H. leucospilota* fisheries has become an increasingly important priority. The complete mitochondrial genome is an excellent molecular marker for studying species identification and genetic diversity. Here, we report the complete mitochondrial genome sequence of *H. leucospilota*, which will provide a useful genetic markers for stock management and genetic assessment.

A tissue samples of *H. leucospilota* from three individuals were collected from GuangXi province, China (Beihai, 20.914499 N, 109.203857 E), and the whole body specimen (#GH0014) were deposited at Marine biological Herbarium, Guangxi Institute of Oceanology, Beihai, China. The total genomic DNA was extracted from the muscle of the specimens using an SQ Tissue DNA Kit (OMEGA, Guangzhou, China) following the manufacturer’s protocol. DNA libraries (350 bp insert) were constructed with the TruSeq NanoTM kit (Illumina, San Diego, CA) and were sequenced (2 × 150bp paired-end) using HiSeq platform at Novogene Company, China. Mitogenome assembly was performed by MITObim (Hahn et al. 2013). complete mitogenome of *Holothuria scabra* (GenBank accession number: NC_027086) was chosen as the initial reference sequence for MITObim assembly. Gene annotation was performed by MITOS (Bernt et al. 2013).

The complete mitogenome of *H. leucospilota* was 15,839 bp in length (GenBank accession number: MN276190), and containing the typical set of 13 protein-coding, 22 tRNA and two rRNA genes, and a putative control region. The overall base composition of the mitogenome was estimated to be A 31.8%, T 25.8%, C 25.8% and G 16.8%, with a high A + T content of 57.6%, which is similar, but slightly lower than *H. scabra* (59.7%) (Xia et al. 2016). The mitogenomic phylogenetic analyses showed that *H. leucospilota* was first clustered with *H. scabra* in the monophyletic Holothuriidae clade (Figure 1), which is consistent with the phylogenetic analyses of family Holothuriidae using rrnL and cox1 genes from mitochondrial DNA (Borrero-Pérez et al. 2010). Our mitogenome data supported the close relationship of *H. leucospilota* and *H. scabra*. The complete mitochondrial genome sequence of *H. leucospilota* will be useful for its genetic research and future conservation and management.

**Figure 1.**
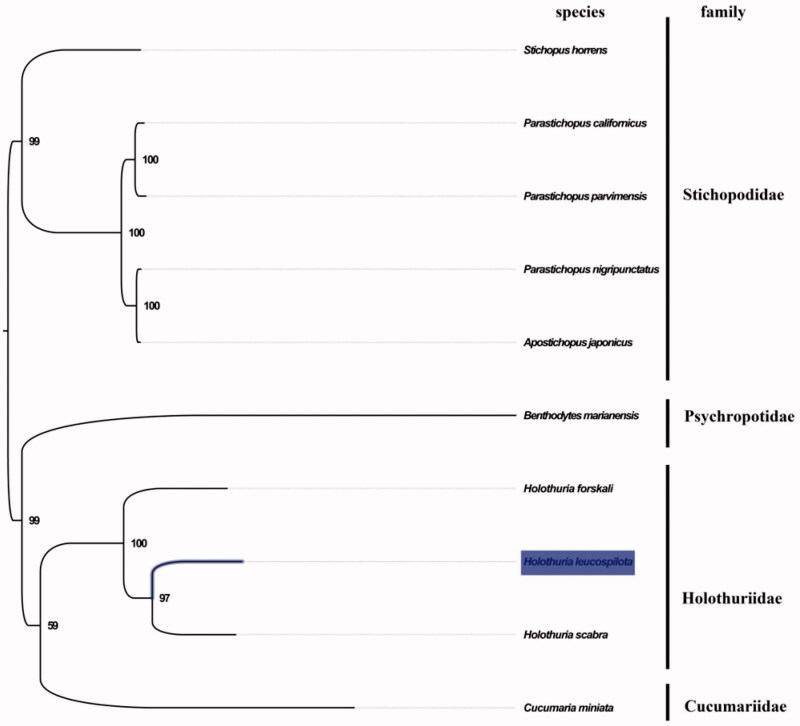
Phylogenetic tree of 10 species in Holothuroidea. The complete mitogenomes is downloaded from GenBank and the phylogenic tree is constructed by maximum-likelihood method with 100 bootstrap replicates. The bootstrap values were labelled at each branch nodes. The gene's accession number for tree construction is listed as follows: *Stichopus horrens* (NC_014454), *Parastichopus nigripunctatus* (NC_013432), *Apostichopus japonicus* (NC_012616), *Parastichopus californicus* (NC_026727), *Parastichopus parvimensis* (NC_029699), *Benthodytes marianensis* (NC_040968), *Holothuria forskali* (NC_013884), *Holothuria scabra* (NC_027086), and *Cucumaria miniata* (NC_005929).
